# Immune response and protective effect against chronic *Toxoplasma gondii* infection induced by vaccination with a DNA vaccine encoding profilin

**DOI:** 10.1186/s12879-018-3022-z

**Published:** 2018-03-07

**Authors:** Qi Gao, Nian-Zhang Zhang, Fu-Kai Zhang, Meng Wang, Ling-Ying Hu, Xing-Quan Zhu

**Affiliations:** 1 State Key Laboratory of Veterinary Etiological Biology, Key Laboratory of Veterinary Parasitology of Gansu Province, Lanzhou Veterinary Research Institute, Chinese Academy of Agricultural Sciences, Lanzhou, Gansu Province 730046 People’s Republic of China; 2 Hunan Entry-exit Inspection and Quarantine Bureau, Changsha, Hunan Province 410004 People’s Republic of China; 3 College of Animal Science, Fujian Agriculture and Forestry University, Fuzhou, Fujian Province 350002 People’s Republic of China; 4 Jiangsu Co-innovation Center for the Prevention and Control of Important Animal Infectious Diseases and Zoonoses, Yangzhou University College of Veterinary Medicine, Yangzhou, Jiangsu Province, 225009 People’s Republic of China

**Keywords:** *Toxoplasma gondii*, Profilin, Immunogenicity, DNA vaccine, Chronic toxoplasmosis

## Abstract

**Background:**

*Toxoplasma gondii* is an obligate intracellular parasite that can infect almost all warm-blooded animals. *T. gondii* profilin (TgPF) plays a crucial role in parasite motility and host cell invasion, and has shown promise against toxoplasmosis. DNA vaccine was considered to elicit effective humoral and cell-mediated immunity against *T. gondii* infection. The objective of the present study was to evaluate the immunogenicity of TgPF in mice using a DNA vaccination strategy.

**Methods:**

A DNA vaccine (pVAX-PF) encoding TgPF gene was constructed and then was intramuscularly injected into mice with and without a plasmid encoding IL-15 (pVAX-IL-15). The immune responses in immunized Kunming mice including lymphocyte proliferation, levels of cytokines, antibody titers and T lymphocyte subclasses were analyzed. The protective efficacy against chronic *T. gondii* infection was observed at 4 weeks post-infection with the cyst-forming PRU strain of *T. gondii* (Genotype II).

**Results:**

EitherpVAX-PF with or without pVAX-IL-15 could elicit higher level of IgG and IgG2a antibodies and produce strong cellular immune responses in the immunized mice. The brain cyst numbers in mice immunized with pVAX-PF + pVAX-IL-15 (1843 ± 215.7) and pVAX-PF (1897 ± 337.8) were reduced 40.82% and 39.08%, respectively, compared to that in mice received nothing (3114 ± 168.8), and the differences were statistically significant (*P* < 0.0001). However, the *T. gondii* cyst numbers in mice immunized with pVAX-PF + pVAX-IL-15 were not statistically significantly different compared to that in mice immunized with pVAX-PF alone [t(10) = 0.33, *P* > 0.05].

**Conclusions:**

The present study indicated that TgPF could be a promising vaccine candidate against chronic toxoplasmosis, which can be further used to develop multi-epitope vaccine formulations in food-producing animals against *T. gondii* infection.

## Background

The obligated intracellular protozoan *Toxoplasma gondii* can infect most all of warm-blooded animals and humans, which would lead to zoonotic toxoplasmosis worldwide [1, 2]. *T. gondii* usually cause subclinical infection in most of immunocompetent adults [3, 4], but the parasite would be a severely risk factor for immunodeficient individuals (HIV-infected patients and transplant recipients), children and pregnancy [5–8]. *T. gondii* is also a common cause of abortion in sheep and goats, leading to serious economic losses [6, 9, 10]. However, no effective treatment was available to eliminate *T. gondii* cysts by now. Immunoprophylaxis against *T. gondii* would be of high priority for the disease control, as previous reviews noted [10–12].

A number of vaccine candidates, including surface antigens (SAG), rhoptry antigens (ROP), microneme antigens (MIC), dense granule antigens (GRA) and some other proteins playing important roles in the life cycle of *T. gondii* have been evaluated against *T. gondii* infection*.* However, no one can completely protect against tissue cysts, usually lower than 80–90% protection [11]. DNA-based vaccines were considered to elicit effective humoral and cell-mediated immunity against *T. gondii* invasion in animal models, which have been used in many previous studies [11–13].

Following the parasite invasion, host immune response is successively suffered innate acute response and an Ag-specific cell-mediated immune response [14]. The invading parasite in mouse model is primarily recognized by Toll-like receptors (TLRs) of DCs, and then triggers the host’s TLRs/MyD88 response [15]. TLR11 and 12 are demonstrated as important receptors for *T. gondii* recognition. Activation of TLR11 and 12 can induce potent cytokine responses, and lack of TLR11 and TLR12 genes, mice were showed rapidly succumb to *T. gondii* infection [16–18]. *T. gondii* profilin (TgPF), one of the ligands of both TLR11 and 12, is essential for the parasite gliding motility, host cell invasion and egress from host cells in mice [17, 19]. TgPF is also shown to be an immunodominant antigen. Immunization of C57BL/6 mice with TgPF encapsulated in oligomannose-coated liposomes induces protective immunity against infection with *T. gondii* tachyzoites (PLK strain) [20].

DNA vaccination can deliver the expressed protein as an endogenous antigen, and has exhibited promise for defense against toxoplasmosis due to the ability of eliciting effective humoral and cellular immune responses in mice [11]. These findings stimulated to hypothesize whether the endogenous TgPF protein could induce effectively protective responses against infection with *T. gondii* tissue cysts, the primary transmission route of *T. gondii* infection for humans [2].

To examine the immunogenicity of the genetic TgPF antigen, we constructed a DNA vaccine encoding TgPF (pVAX-PF), and used a plasmid encoding murine costimulatory molecule IL-15 (pVAX-IL-15) as genetic adjuvant. The pVAX-PF DNA vaccine with or without pVAX-IL-15 were examined for their ability of eliciting immune responses and their protective efficacy against chronic *T. gondii* infection in a murine model.

## Methods

### Mice and parasites

A total of 108 specific-pathogen-free (SPF) grade female Kunming mice aged six to eight weeks were purchased from Lanzhou University Laboratory Animal Center (Lanzhou, China). All mice were handled in strict accordance with good animal practices according to the Animal Ethics Procedures and Guidelines of the People’s Republic of China.

Tissue cysts of the low virulent PRU strain of *T. gondii* (Genotype II) were preserved in State Key Laboratory of Veterinary Etiological Biology, Lanzhou Veterinary Research Institute, Chinese Academy of Agricultural Sciences, Lanzhou, Gansu Province, China. The cysts of the PRU strain were obtained from the brains of orally infected Kunming mice one month after intragastric administration of the cysts.

### Expression of TgPF protein in *Escherichia coli*

Total RNA was extracted from *T. gondii* bradyzoites using E.Z.N.A. Total RNA Kit I (Omega, America). The complete open reading frame (ORF) of TgPF gene was amplified by reverse transcription-polymerase chain reaction (RT-PCR) using a pair of specific primers (prfF: 5′- CGG GGTACC ATGTCCGACTGGGACCCTGTTGTCAAGG -3′, prfR: 5′- CCG GAATTC TTAGTACCCAGACTGGTGAAGATACTCG - 3′), designed according to the corresponding sequence of the ME49 strain (ToxoDB: TGME49_293690), in which the *Kpn* I and *Eco*R I restriction sites were introduced and underlined. The RT-PCR was performed following the instruction of PrimeScript® One Step RT-PCR Kit Ver.2 (TaKaRa, China). Briefly, the program was initiated at 50 °C for 30 min to synthesize the first strand cDNA, followed by 35 cycles of 94 °C for 35 s (denaturation), 65.5 °C for 45 s (annealing), 72 °C for 50 s (extension) and a final extension of 72 °C for 10 min.

The obtained DNA was ligated into pET-30a(+), and then transformed into *E. coli* BL21 (DE3) strain. The positive recombinant plasmids were identified by both restriction enzyme digestion using *Kpn* I and *Eco*R I and sequencing (Sangon, China). The recombinant TgPF (rTgPF) protein was expressed at the condition of 0.8 mmol/L IPTG (Sangon, China), shaking for 8 h at 37 °C. The rTgPF protein was purified using Ni-NTA His bind resin (Novagen) according to the manufacturer’s instructions and were visualized by the sodium dodecyl sulfate-polyacrylamide gel electrophoresis (SDS-PAGE).

### Construction of the eukaryotic expression plasmids

The obtained TgPF gene and the eukaryotic expression plasmid pVAX I (Invitrogen, USA) were digested by restriction enzymes, *Kpn* I and *Eco*R I, respectively. After purification of the two DNA fragments using the Universal DNA Purification Kit (TIANGEN, China), the TgPF gene was linked to pVAX I by T4 DNA ligase (TaKaRa, China). After identification by sequencing, the positive recombinant plasmid pVAX-TgPF was constructed. The pVAX-IL-15 plasmids were preserved in our laboratory as described previously [21], and were identified by sequencing before use (Sangon, China). Both of the two eukaryotic plasmids were transformed into *E. coli* DH5α and then were purified by anion exchange chromatography (EndoFree Plasmid Giga Kit, Qiagen Sciences, MD, USA) following the manufacturer’s instructions and stored at − 20 °C until use. The concentrations of the two plasmids were determined by spectrophotometer at OD260 and OD280.

### Expression of pVAX-PF in vitro

HEK293 cells were transfected with the pVAX-PF or the empty pVAX I (negative control) using lipofectamine™ 2000 reagent (Invitrogen) following the manufacturer’s instructions. The expression of pVAX-PF plasmid was examined by the indirect immunofluorescence assay (IFA) at 48 h after transfection. Briefly, the HEK293 cells were fixed with acetone and then perforated with 0.1% Triton-X-100 in PBS. The cells were incubated with goat polyclonal antibodies against *T. gondii* tachyzoites (1: 50 in PBS) at 37 °C for 60 min, followed by fluorescein isothiocyanate (FITC)-labeled anti-goat IgG antibodies (Abcam, UK) diluted 1: 2000 in PBS. The specific fluorescence was imaged using a Zeiss Axioplan fluorescence microscope (Carl Zeiss, Germany).

### Immunization and challenge

Mice were inoculated intramuscularly at bilateral quadriceps with 100 μg of pVAX-PF + pVAX-IL-15 (group I), 100 μg of pVAX-PF (group II), 100 μg of pVAX-IL-15 (group III), 100 μg of pVAX I (group IV), or sterile phosphate buffered saline (PBS) alone (group V) (each 100 μl) for three times at two-week interval. Mice which were not inoculated constituted the blank control (group VI). There were 18 mice in each group. Mice from group III, group IV, group V and group VI were treated as controls. Two weeks after the last immunization, splenocytes were aseptically harvested and the red blood cells were removed. All analyses were performed in triplicate.

To measure the tissue cyst burden in mice, 2 weeks after the third vaccination, six mice in per group were inoculated orally with 10 tissue cysts of the PRU strain and the brain cysts were determined at 4 weeks post-infection.

### Determination of antibody titers and isotype

Blood samples were collected from mice in each group at 0, 2, 4 and 6 weeks from the tail vein for analysis of specific antibodies. The IgG subclasses (IgG1 and IgG2a) were examined using the sera collected at 6 weeks. The levels of TgPF-specific immunoglobulin G (IgG), IgG1 and IgG2a were measured by ELISA using SBA Clonotyping System-HRP Kit (Southern Biotech CO., LTD, Birmingham, USA) following the manufacture’s instruction. Briefly, the purified rTgPF protein (5 μg/mL) was coated on the 96-well plates over night at 4 °C. After blocking the non-specific sites by 0.5% BSA in PBS, the wells were incubated with 100 μL of mouse serum sample from each group at 37 °C for 1 h. Then, the horseradish-peroxidase (HRP) conjugated anti-mouse IgG antibodies (1: 2500 dilutions, 100 μL), or anti-mouse IgG1 or IgG2a (1: 500 dilutions, 100 μL) were added into each well. The absorbance of each well was examined at 450 nm after incubating with substrate solution (pH 4.0) (1.05% citrate substrate buffer; 1.5% ABTS; 0.03% H_2_O_2_) in the dark for 20 min.

### Lymphocyte proliferation assay by MTs

The harvested splenocytes from each mouse were cultured in triplicate at a density of 2 × 10^5^ cells per well in complete medium (DMEM medium + 10% FCS + 100 U/mL penicillin/streptomycin). The cells from each group were stimulated with rTgPF (10 μg/mL) and medium alone, respectively, at 37 °C in a 5% CO_2_ incubator. The proliferative activity was measured using MTs method (Promega, USA) after four days. After examining the OD_570_ value of wells, the stimulation index (SI) was calculated using the formula OD_570TLA_/OD_570M_.

### Flow cytometry analysis

The concentration of the purified splenocytes were adjusted into 1× 10^5^ cells/mL. Then the phycoerythrin (PE)-labeled anti-mouse CD3 (eBioscience) (5 μg/mL), Allophycocyanin (APC)-labeled anti-mouse CD4 (eBioscience) (5 μg/mL) and fluorescein isothiocyanate (FITC)-labeled anti-mouse CD8 (eBioscience) (5 μg/mL) antibodies were used to stain the T cell subclasses (CD4+ and CD8+) for 30 min at 4 °C. After washing by PBS, the cultures were fixed with FACScan buffer (1% FCS and 0.1% Sodium azide in PBS) and 2% paraformaldehyde. The T cell subclasses were measured for fluorescence profiles on a FACScan flow cytometer (BD Bio-sciences) and analyzed by SYSTEM II software (Coulter).

### Cytokine assays

To further evaluate the role of the DNA vaccination, the levels of cytokines including IL-2, IL-4, IL-10 and IFN-γ was carried out. Briefly, the purified splenocytes of mice from each group were co-cultured with rTgPF as positive control and medium alone as negative control at 24 h for IL-2 and IL-4, 72 h for IL-10, and 96 h for IFN-γ. The supernatant was evaluated to the concentration of each cytokine using the commercial ELISA kits (Biolegend, USA). 50 μL of Assay Buffer A and equal volume of the supernatant of each cell culture were successively added in to microplate well, incubating for 2 h at room temperature. Then, 100 μL of detection solution was added into each well. After washing the plate for 4 times, Avidin-HRP A solution was added and incubated for 30 min, and subsequently the substrate solution E was added. The reaction was stopped after adding 100 μL of stop solution into each well. The absorbance was read at 450 nm. The sensitivity limits for the assays were 20 pg/ml for IFN-γ, 10 pg/ml for IL-4 and IL-10, and 50 pg/ml for IL-2, respectively. The assay was performed in three independent experiments.

### Statistical analysis

All statistical analyses were performed following the procedure of GraphPad Prism 5.0. The differences of the data regarding antibody responses, lymphoproliferation assays, percentages of CD4+ and CD8+ T cells, cytokine production and brain cyst loading were compared by one-way ANOVA. The difference compared in pairs was calculated by the *t*-test. The results in comparisons between groups were considered different if *P* < 0.05.

## Results

### Sequencing results of the pVAX-PF and pVAX-IL-15

After identification by restriction enzyme digestion, the positive pVAX-PF plasmids were then confirmed by sequencing. The resulting sequence of pVAX-PF plasmids had 100% nucleotide sequence identity with the corresponding sequence of the RH strain (GenBank accession No.: AK223678.1). Sequencing of pVAX-IL-15 showed that no base deletion or change was detected after alignment with the corresponding sequence in GenBank (accession no. NM_008357.1).

### Expression of pVAX-PF in vitro

**Fig. 1:**
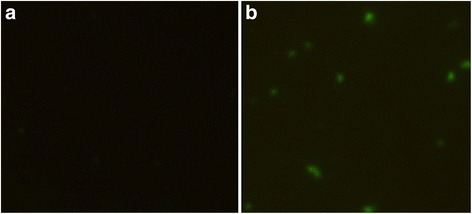
Analyses of expression of TgPF gene on HEK293 cells by indirect immunofluorescence (IFA) at 48 h post-transfection. **a** empty vector pVAX I; **b** HEK293 cells transfected with pVAX-PF

The expression of pVAX-PF was identified by IFA. Cells transfected with the eukaryotic recombinant plasmid pVAX-PF showed specific green fluorescence. However, there was no green dot in the cells transfected with the same amount of pVAX I (Fig. 1).

### Evaluation of the humoral immune responses induced by DNA immunization

**Fig. 2:**
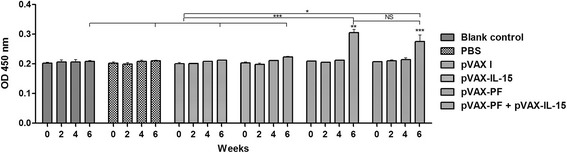
Determination of IgG antibodies in the sera of Kunming mice at 0, 2, 4, and 6 weeks. Mice immunized with pVAX-IL-15, pVAX I, PBS and nothing were treated as controls in statistical analysis. Each bar represents the mean OD (±S.E., *n* = 3). ***P < 0.0001, ** *P* < 0.01, * *P* < 0.05, NS: not significant

The significantly increased anti-TgPF IgG antibodies after the last booster were detected only in the mice immunized with pVAX-PF [*t*(3) = 9.721, *P* < 0.01] or pVAX-PF + pVAX-IL-15 [*t*(3) = 13.99, *P* < 0.0001]. Two weeks after the last immunization, the antibody levels in mice immunized with pVAX-PF (*P* < 0.01) and pVAX-PF + pVAX-IL-15 (*P* < 0.05) were significantly increased than those in the control groups (Fig. 2). Mice from group II produced a slightly higher level of IgG antibody after the third vaccination compared to that from group I, but the difference was not significant (*P* > 0.05).

### Examination of the types of immune response

**Fig. 3:**
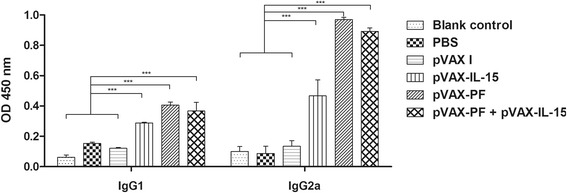
Analysis of IgG isotypes, IgG1 and IgG2a, in the sera of Kunming mice 2 weeks after the last immunization. Mice immunized with pVAX I, PBS and nothing were treated as controls in statistical analysis. Each bar represents the mean OD (±S.E., *n* = 3). ***P < 0.0001

The IgG isotypes were examined to determine whether a Th1 or Th2 response was elicited in the immunized mice by DNA vaccination. As shown in Fig. 3, both IgG1 and IgG2a antibodies in the sera of mice from group I, II and III were significantly increased at 2 weeks after the last immunization, with higher levels of IgG2a to IgG1. These results indicated that pVAX-IL-15, pVAX-PF and pVAX-PF + pVAX-IL-15 are able to elicit a Th1 type immune response.

### Analysis of cellular immune responses

As shown in Table 1, splenocytes from mice in Group I [F(4,10) = 4.531, *P* < 0.01] and Group II [F(4,10) = 7.675, *P* < 0.01] were significantly increased after stimulating with rTgPF compared with that in mice from the control groups. The difference in SI between Group I and Group II was not statistically significant [t(4) = 1.823, *P* > 0.05].

**Table 1:** Splenocyte proliferative responses and the percentages of T cell subsets in immunized mice 2 weeks after the last immunization

Groups	SI (Mean ± SD)	CD3 + CD4+ (%)	CD3 + CD8+ (%)
pVAX-PF + pVAX-IL-15	1.08 ± 0.06^A^	11.13 ± 3.02^A^	7.13 ± 1.23^A^
pVAX- PF	1.13 ± 0.11^A^	11.33 ± 3.23^A^	8.9 ± 1.13^A^
pVAX-IL-15	0.85 ± 0.05^B^	14.53 ± 1.17^A^	6.43 ± 2.32^A^
pVAX I	0.79 ± 0.03^B^	3.13 ± 1.36^B^	5.77 ± 2.6^A^
PBS	0.85 ± 0.04^B^	3.46 ± 1.25^B^	5.77 ± 0.67^A^
Blank control	0.56 ± 0.01^C^	6.43 ± 1.42^B^	6.1 ± 1.82^A^

Spleens from 3 mice in each group

SI stands for stimulation index

The same superscript letter means no significant difference (*P* > 0.05), whereas different superscript letters mean significant difference (*P* < 0.05)

The T cell subclasses of mice immunized with various vaccinations were identified by flow cytometry analysis. Percentages of CD3+ CD4+ T lymphocytes from Group I [F(3,8) = 11.28, *P* < 0.01], Group II [F(3,8) = 10.87, *P* < 0.01] and Group III [F(3,8) = 49.51, *P* < 0.0001] were significantly higher than those in mice received pVAX I, PBS or nothing, and were not statistically different among mice in the three control groups. The ratios of CD3+ CD8+ T cells in the spleens of mice vaccinated with pVAX-PF [F(4,10) = 1.519, *P* = 0.27] and pVAX-PF + pVAX-IL-15 [F(4,10) = 0.28, *P* = 0.88] was slightly higher than that in all control groups, but the difference was not statistically significant.

### Cytokine production by spleen cells

**Fig. 4:**
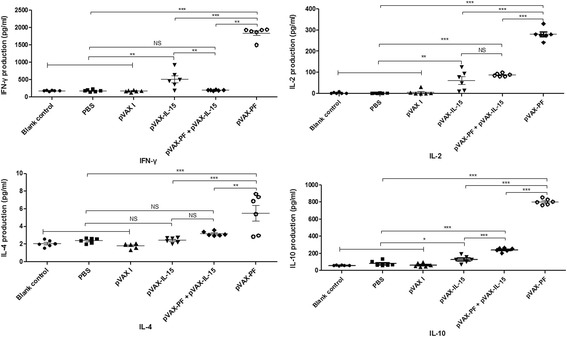
Cytokine productions secreted by splenocytes of Kunming mice immunized after stimulating by rTgPF protein. Each bar represents the mean OD (±S.E., *n* = 6). ***P < 0.0001, ** *P* < 0.01, * *P* < 0.05, NS: not significant

As shown in Fig. 4, splenocytes in mice from Group I induced significantly higher levels of IL-2 (*P* < 0.001) and IL-10 (*P* < 0.01) compared to that in mice from Group IV, V and VI, but the levels of IFN-γ (*P* > 0.05) and IL-4 (*P* > 0.05) were not significantly different. The levels of IFN-γ, IL-2, IL-4 and IL-10 in spleen cell cultures from pVAX-PF immunized mice were significantly higher than that in the control groups (*P* < 0.01). Mice immunized with pVAX-PF induced significantly higher levels of IFN-γ (*P* < 0.01), IL-2 (*P* < 0.001), IL-4 (*P* < 0.01) and IL-10 (*P* < 0.0001) than that in mice immunized with pVAX-PF + pVAX- IL-15.

### Assessment of protective activity

**Fig. 5:**
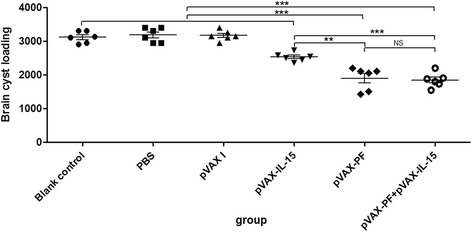
Protection against chronic toxoplasmosis in mice immunized with pVAX-PF + pVAX-IL-15, pVAX-PF alone and in controls two weeks after the last booster. Mice immunized with pVAX-IL-15, pVAX I, PBS or nothing were treated as controls in statistical analysis. Each bar represents the mean number (±S.E., *n* = 6). *** *P* < 0.0001. ** *P* < 0.01. NS: not significant

To evaluate whether pVAX-PF with or without pVAX-IL-15 could induce sufficient protection against the formation of *T. gondii* tissue cysts in brain, mice of all the groups were challenged with 10 cysts of *T. gondii* PRU strain and the brain cyst loadings were assessed 28 days after challenge. The number of tissue cysts in mice of Group I (1843 ± 215.7) and group II (1897 ± 337.8) were reduced 40.82% and 39.08%, respectively, compared to that in mice received nothing (3114 ± 168.8). Immunization with pVAX-PF + pVAX-IL-15 [F(4,25) = 64.53, *P* < 0.0001] and pVAX-PF alone [F(4,25) = 42, *P* < 0.0001] significantly decreased the brain cyst formation in mice compared with all the control groups (Fig. 5). The brain cyst numbers in mice from Group I were slightly lower than that from Group II, but the difference was not statistically significant [t(10) = 0.33, *P* > 0.05]. Brain cyst loadings in mice immunized with pVAX-IL-15 were significantly lower compared to that in mice vaccinated with pVAX-PF + pVAX-IL-15 [t(10) = 6.73, *P* < 0.0001] and pVAX-PF [t(10) = 4.30, *P* < 0.01], but were significantly higher than that in mice received pVAX I, PBS or nothing [F(3,20) = 21.11, *P* < 0.0001].

## Discussion

In the present study, DNA vaccination of mice with pVAX-PF with or without pVAX-IL-15 could elicit strong humoral and cellular immune responses. The protective activity showed that significant brain cyst reductions were found in mice immunized with pVAX-PF and pVAX-PF + pVAX-IL-15 compared to that in controls, which suggested that the endogenous TgPF protein could strongly induce protective effect against chronic toxoplasmosis. To avoid missing inspection, each sample was examined for 5 times by microscopy. At each time, 10 μl of suspensions were dripped onto glass slide, and were checked by three different persons.

IFN-γ production and cytotoxic activity of CD8+ T cells play important roles for *T. gondii* clearance [22]. The specific CTLs, main subsets of Th1 cells, can directly destroy the host cells containing intracellular niches of *T. gondii* or through secretion of IFN-γ [23]. Another important Th1-skew cytokine for the *T. gondii* resistance is IL-2, which can regulate not only proliferation but also activities of CTLs through an autocrine feedback loop after antigen stimulation [24, 25]. The adaptive immune response is thus induced toward Th1 type [14, 26]. In the present study, significantly increased levels of IFN-γ and IL-2 were detected in mice immunized with pVAX-PF, suggesting induction of a Th1 type immune response, which contributed to the lower brain cyst numbers. Further study should be investigated on the CD4+ and CD8+ T cells excreting cytokines, such as INF-γ or IL-12.

Previous studies have shown that IL-15 can prolong the duration of CD8 + T cell-mediated immunity [27–29]. Immunizations with IL-15 as adjuvant showed to improve the protective efficacy of vaccine candidates against *T. gondii* infection in Kunming mice [21, 30]. Herein, DNA immunization with pVAX-IL-15 can also facilitate specific humoral and cellular immune responses, which further indicated the potential of IL-15 as adjuvant against chronic toxoplasmosis through prolonging the survival of memory T cells [27].

However, the *T. gondii* cyst number in mice immunized with pVAX-PF + pVAX-IL-15 was not significantly different compared to that in mice from Groups II and III. The humoral and cellular immune responses, such as levels of IgG, IFN-γ, IL-2 and the percent of CD8+ T cells, in mice immunized with pVAX-PF + pVAX-IL-15 were weak than that in mice immunized with pVAX-PF, which were different to our previous studies using other antigens [21, 30]. IL-15 is structural similar to IL-2, and some subunits of the IL-15 receptor are shared in common with the receptor of IL-2. Whether the weak effect of co-immunization of pVAX-PF and pVAX-IL-15 is resulting from negative regulation of mutual activities through IL-15 completing the common receptors with IL-2 evoked by pVAX-PF should be further studied.

Specific antibody could attach the parasite to the host cell receptors or the complement molecular. The humoral responses thus are also considered to play an important role in immunity against *T. gondii* [31]. Herein, the specific IgG antibodies were firstly identified by western blotting after the purified rTgPF separated by SDS-PAGE (data not shown). The enhanced humoral immune response including IgG and IgG2a antibodies in mice immunized with pVAX-PF + pVAX-IL-15 or pVAX-PF would be essential for the decrease of brain cyst number. The results were consistent with our previous studies vaccinating mice with plasmids coding CDPK1 [30], ROM4 and ROM5 [32], or immunizing with subunit vaccine [33].

## Conclusions

In conclusion, the present study indicated that immunization with DNA vaccine encoding pVAX-PF with or without pVAX-IL-15 could significantly decrease the brain cyst loadings established in Kunming mice compared to controls, with higher level of IgG and IgG2a antibodies and a strong cellular immune response, which suggested that TgPF would be a good candidate for development of vaccines against *T. gondii* cyst formation used in food-producing animals.
